# Environmental factors regulating gaping activity of the bivalve *Arctica islandica* in Northern Norway

**DOI:** 10.1007/s00227-017-3144-7

**Published:** 2017-04-27

**Authors:** Irene Ballesta-Artero, Rob Witbaard, Michael L. Carroll, Jaap van der Meer

**Affiliations:** 1 Department of Coastal Systems, NIOZ; Netherlands Institute for Sea Research and Utrecht University, PO Box 59, 1790 AB Den Burg, Texel, The Netherlands; 2 Department of Estuarine and Delta Systems, NIOZ; Netherlands Institute for Sea Research and Utrecht University, PO Box 140, 4400 AC Yerseke, The Netherlands; 3 Akvaplan-niva, FRAM-High North Centre for Climate and the Environment, 9296 Tromsø, Norway

## Abstract

*Arctica islandica* is the longest-living non-colonial animal known at present. It inhabits coastal waters in the North Atlantic and its annual shell increments are widely used for paleoclimatic reconstructions. There is no consensus, however, about the intra-annual timing of its feeding activity and growth. This research aims to identify the main environmental drivers of *A. islandica* valve gape to clarify the ambiguity surrounding its seasonal activity. A lander was deployed from February 2014 to September 2015 on the sea bottom at Ingøya, Norway (71°03′N, 24°05′E) containing living *A. islandica* specimens (70.17 ± 0.95 mm SE) in individual containers. Each individual was attached to an electrode unit that measured the distance between their valves (valve gape) every minute. Individuals were followed for various lengths of time, and in some cases replaced by smaller individuals (54.34 ± 0.63 mm SE). The lander was also equipped with instruments to simultaneously monitor temperature, salinity, [Chl-a], turbidity, and light. There was a significant difference in the average monthly valve gape (*P* value < 0.01), with monthly means of 19–84% of the total valve gape magnitude. The experimental population was largely inactive October–January, with an average daily gape <23%. During this period the clams opened at high amplitude once or twice a month for 1–3 days. Seasonal cycles of sea water temperature and [Chl-a] were temporally offset from each other, with temperature lagging [Chl-a] by about 2 months. Multiple regression analyses showed that bivalve gaping activity was most closely correlated with variable [Chl-a], and to a much smaller degree with photoperiod and temperature.

**Electronic supplementary material:**

The online version of this article (doi:10.1007/s00227-017-3144-7) contains supplementary material, which is available to authorized users.

## Introduction

The bivalve *Arctica islandica*, also known as the ocean quahog, inhabits coastal waters in the North Atlantic (Jones 1980; Dahlgren et al. 2000). The species is the longest-living non-colonial animal yet known, with a longevity of >500 years (Butler et al. 2013). As in other bivalves, a history of their growth is retained in their shells. Shell growth increments (or growth bands) can provide basic biological information on the species including age and growth rate. Moreover, the pattern of these bands and the composition of the shell material therein can reflect environmental conditions when the shell was deposited.

Annual synchronization of band widths among individuals in a population has been identified in numerous studies (Jones 1980; Witbaard et al. 1997a; Butler et al. 2009; Mette et al. 2016), suggesting that synchronous shell growth is influenced by a common environmental signal (Marchitto et al. 2000; Schöne et al. 2003; Dunca et al. 2009; Marali and Schöne 2015). Temperature alone does not always fully explain variations in growth performance (Witbaard et al. 1996). Food availability is considered important in explaining the various reports on its growing season (Witbaard et al. 1996; Schöne et al. 2003; Witbaard et al. 2003). In the Fladen Ground (North Sea), for instance, an eddy system led to the import and accumulation of organic matter into that area (Witbaard et al. 1996), and this hydrodynamic feature was identified as the factor responsible for the growth variation of *A. islandica* there.

Likewise, high synchrony in valve gape, i.e., the distance between a valve pair, has been observed in various bivalve species, also suggesting that a common external force with a periodicity similar to gaping drives the response (Thorin 2000; Borcherding 2006; Mat et al. 2012; García-March et al. 2016). Based on those studies, food, temperature, and light conditions are considered key drivers of valve activity. Earlier studies on *A. islandica* (Winter 1969) and other bivalves (Higgins 1980; Williams and Pilditch 1997; Riisgård et al. 2006) identified the presence of Chl-a as the main driver for a sustained opening of their valves. Other studies, however, demonstrated that light conditions can directly trigger valve gape activity of species such as *Pinna nobilis* and *Hippopus hippopus* (García-March et al. 2008; Schwartzmann et al. 2011).

The confounded roles of temperature, light and food in regulating activity patterns and shell growth in *A. islandica* can be disentangled most effectively in populations occurring near their biogeographical limits, where small variations in environmental conditions can have large impacts on physiological functions and performance. In the present study, we analyzed *A. islandica* gaping activity patterns in relation to key environmental factors in an Arctic region in Northern Norway. The light cycle at this latitude (71°N) exhibits extreme seasonal variations in light intensity and day length (Kaartvedt 2008), and could have a major influence on the seasonal gaping activity of *A. islandica*.

Filter-feeding bivalves must open their valves and extend their siphons to filter water, to respire and feed. Thus, wide open valves indicate periods of feeding and respiration (Møhlenberg and Riisgård 1979; Newell et al. 2001; Riisgård and Larsen 2015). In contrast, the reduction of the opening distance or total closure of valves implies a retraction of the mantle edges and siphons, resulting in a reduction and eventually in a cessation of filtration (Møhlenberg and Riisgård 1979; Riisgård and Larsen 2015). Witbaard et al. (1997b) measured siphon activity in *A. islandica* juveniles as the number of times an individual had the mantle extended with open siphons. It was expressed as the percentage of the total number of observations per specimen and then they calculated an average of siphon activity for multiple individuals per treatment. Siphon activity varied from 12% in a treatment with no food to 76% in the highest food concentration. This study thus also demonstrated a positive relationship between high siphon activity and growth in all treatments (Witbaard et al. 1997b). These results suggest that valve opening and closing of *A. islandica* can be used as a proxy for active feeding and as an indicator of periods of potential growth.

Based on those earlier lab experiments (Møhlenberg and Riisgård 1979; Witbaard et al. 1997b; Newell et al. 2001; Riisgård and Larsen 2015), we designed an in situ experiment to link gaping activity to environmental factors. We set up a field study of *A. islandica* at its northern geographical limit (Dahlgren et al. 2000; Mette et al. 2016) to examine environmental regulation of shell gaping activity. Locally collected living individuals of *A. islandica* with an electrode array attached to their valves were deployed on the sea bottom for various lengths of time in the period February 2014–September 2015. Valve gaping activity was measured together with environmental conditions (temperature, salinity, [Chl-a], turbidity, and light) to provide insight into environmental factors controlling seasonal changes in *A. islandica*.

## Methods

### Site description

**Fig. 1:**
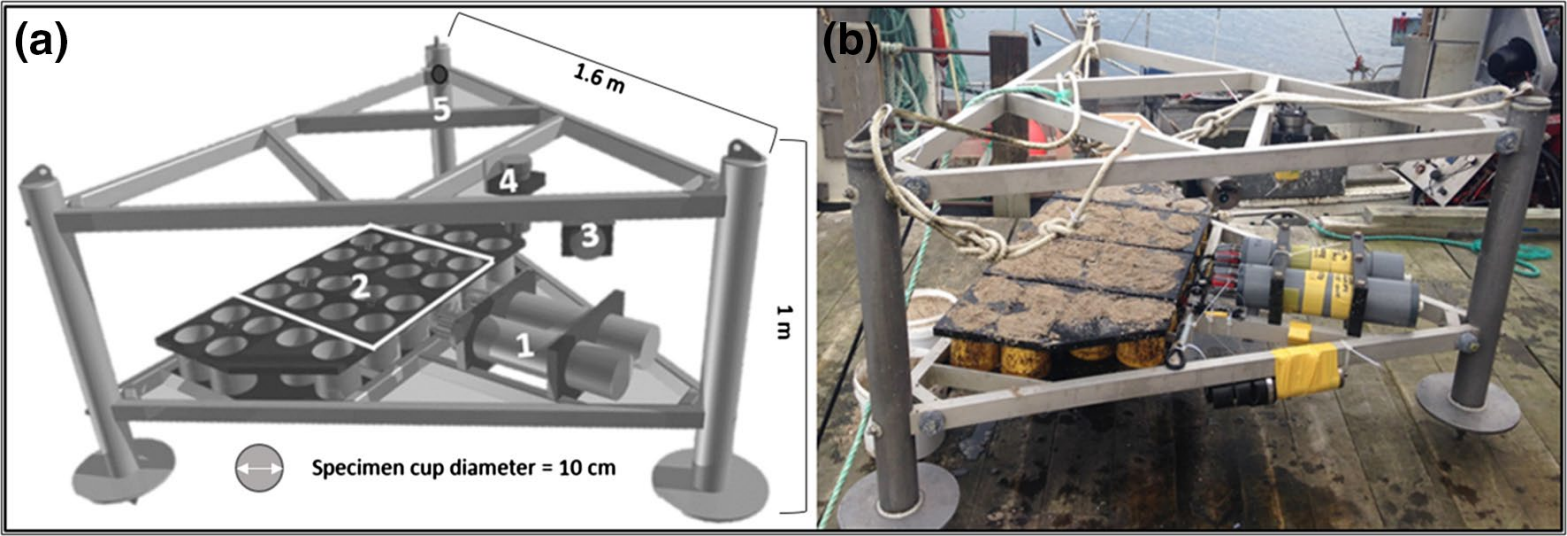
**a** Benthic lander details (*triangular* aluminum frame): *1*. Valve gape recorders, *2*. Specimen cups: 16 for valve gape the experiment (within *white rectangle*), and 12 controls for future growth studies, *3*. ALEC fluorimeter, *4*. ALEC PAR sensor, *5*. Conductivity and temperature. **b** Benthic lander before deployment

The in situ experiment took place at a 10-m deep site in Sanden Bay (71°03′N, 24°05′E), on the east side of Ingøya (Finnmark, northern Norway; Fig. 1 Online Resource 1). Ingøya is located ~15 km northwest of the Norwegian mainland and 60 km west of North Cape. Sanden Bay is exposed to the open Barents Sea from the northeast but protected from the full oceanic swell by a series of islets at its mouth. The seafloor in the bay is a mosaic of rocky outcrops covered with kelp, intermixed with patches of shell sand and maerl-like soft sediments with a median grain size of 400 µm (silt <1%). *Arctica islandica* densities of ~10 m^−2^ occur within these patches of soft sediments. This Sanden Bay population appears to be the northern most known (Dahlgren et al. 2000; Mette et al. 2016).

Ingøya is located ~480 km north of the Arctic Circle, where there is almost complete darkness, October–mid-February. Due to rapidly increasing light levels in the spring, phytoplankton growth starts at the end of March when sea water temperatures are still at their coldest (Carroll et al. 2009). In temperate zones, temporal patterns of temperature and phytoplankton growth are usually strongly linked and distinguishing their impacts on activity or growth is not easy. However, in northern Norway, sea temperature lags primary productivity by ~2 months.

### Lander description

For the in situ experiment, we used a benthic lander constructed at the Royal Netherlands Institute for Sea Research (Texel, Netherlands) (Fig. 1). The lander weighs around ~150 kg, with lead-weighted legs to ensure stability on the sea bottom. It consists of a triangular aluminum frame (Al 50St) with sides of 1.6 m and height of 1 m. This structure was used as a platform for the various instruments. We used two valve gape monitors with 8 specimen cups each and an array of environmental sensors including a CT (conductivity and temperature sensor), a turbidity/fluorescence and a light sensor (Fig. 1). The lander was placed on the seafloor by slowly lowering it with a line from the ship. A buoy marked the position for later retrieval.

### Valve gape monitors

Two valve gape monitor control units were attached to the lower triangular part of the lander frame (Fig. 1), 30 cm above the seafloor when deployed. Each valve gape monitor consists of a waterproof PVC housing with eight pairs of electro-coils coated by plastic tubing (1 mH coils with self-extinguishing polyolefin sleeve; http://www.te.com/usa-en/home.html). The electro-coils are connected to an electronic circuit board with 0.6-mm coaxial cable. The electrical circuit board contains a 2-GB SD memory card to store the data. The system is powered by a 5-V battery pack.

Each valve gape monitor unit simultaneously records the valve gape, or distance between the two valves, of eight individual *A. islandica* specimens. Valve distance is defined as the space between the two paired valves at the siphon end. The distance is measured by the electro-coils based on electromagnetic theory. The electro-coils were glued on the siphon side of each valve with light-curing dental resin cement (3M ESPE RelyX Unicem 2Clicker).

Each clam was placed in an individual PVC cylinder, or specimen cup, filled with local sand. The specimen cups were 15 cm high with a diameter of 10 cm. The largest specimen had a shell height of 7.8 cm and length of 8.8 cm. Thus, the specimens had enough space to maneuver in their cups and orient themselves. Each cup had a perforated bottom to allow entry of the wires with coils from the PVC valve gape monitor housing. The wires were long enough to allow movement of the individuals within the cup.

The valve gape monitors were programmed so that once a minute the active coil generated an electromagnetic field which resulted in a current in the second responsive coil. The strength of the measured electromagnetic field (electrical signal) depends on the distance between the two coils and thus the distance between the valves (valve gape).

The raw electrical signal data were amplified and stored on the internal memory card for later conversion into a distance measurement. A calibration indicated that the distance between the two coils could be determined by the following linear relationship:

$$D_{t} \propto \sqrt {\left( {\frac{1}{{S_{t} }}} \right)} ,$$

where $D_{t}$ equals the distance in mm between the coils at time ($t$), and $S_{t}$ is the electrical signal strength at time ($t$).

The coils could not be fixed to the shell at exactly the same distance from the valve edge or from each other in each specimen. To make the results comparable between individuals and periods, the measured distance was recalculated for each individual separately and expressed as a relative valve gape, hereafter called valve gape ($G_{t}$):

$$G_{t} = \frac{{ D_{t} - \hbox{min} \left( {D_{t} } \right)}}{{\hbox{max} \left( {D_{t} } \right) - { \hbox{min} }\left( {D_{t} } \right)}}.$$

With this convention, ($G_{t}$) varies between 0 (fully closed) and 1 (fully open valves).

We made measurements over 592 days, February 2014–September 2015, excluding short periods of 1–3 days when the lander was recovered for servicing (Table 1). A total of 21 individuals were monitored during four periods, including one for the entire time, ten for the first three periods, and seven for at least two consecutive periods. The resulting time series of valve gape data thus spanned 20 months with a maximum of 1440 data points day^−1^ specimen^−1^.

**Table 1:** Deployment periods with specimen identity number by channel

Deployments	D1 (110 days)	D2 (82 days)	D3 (233 days)	D4 (161 days)
Starting day	7/Feb/2014	29/May/2014	22/Aug/2014	15/Apr/2015
Ending day	28/May/2014	19/Aug/2014	12/Apr/2015	9/Sep/2015
Channel 1	B649	B649	B649	
Channel 2	B655	B655	B655	
Channel 3	B658	B658	B658	
Channel 4	B660	B660	B660	
Channel 5	B661	B661	B661	
Channel 6	B665	B665	B665*	
Channel 7	B666	B666	B666	
Channel 8	B667	B667	B667	
Channel 9	B668	B668	B668	B682
Channel 10	B669	B669	B669	B683
Channel 11	B670		B670	B684
Channel 12	B671	B671	B671	B671
Channel 13	B672	B672		
Channel 14			B677	B677
Channel 15	B674	B674	B678	B678
Channel 16	B675	B675	B680	B680

**Fig. 2:**
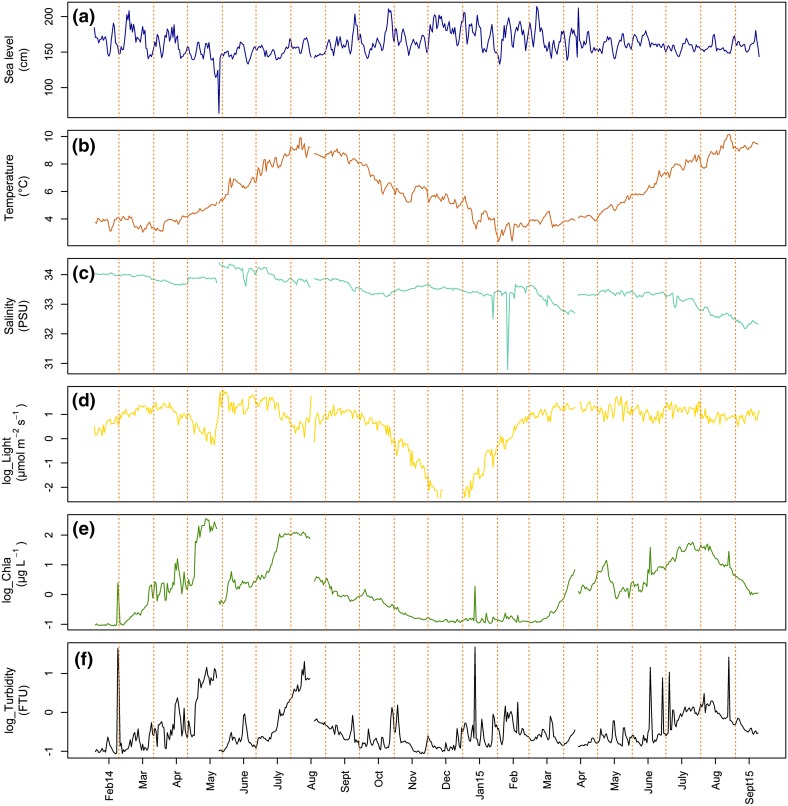
Daily averaged time series of the measured environmental variables by month. **a** Sea level (cm); **b** Temperature (°C); **c** Salinity (PSU); **d** Light conditions (log_µmol m^−2^ s^−1^); **e** [Chl-a] (log_µg L^−1^); **f** Turbidity (log_FTU). Calendar years and months are indicated along *horizontal axis* (14 = year 2014, and 15 = year 2015). Some sensors were fouled prior to lander retrieval in May 2014

**Fig. 3:**
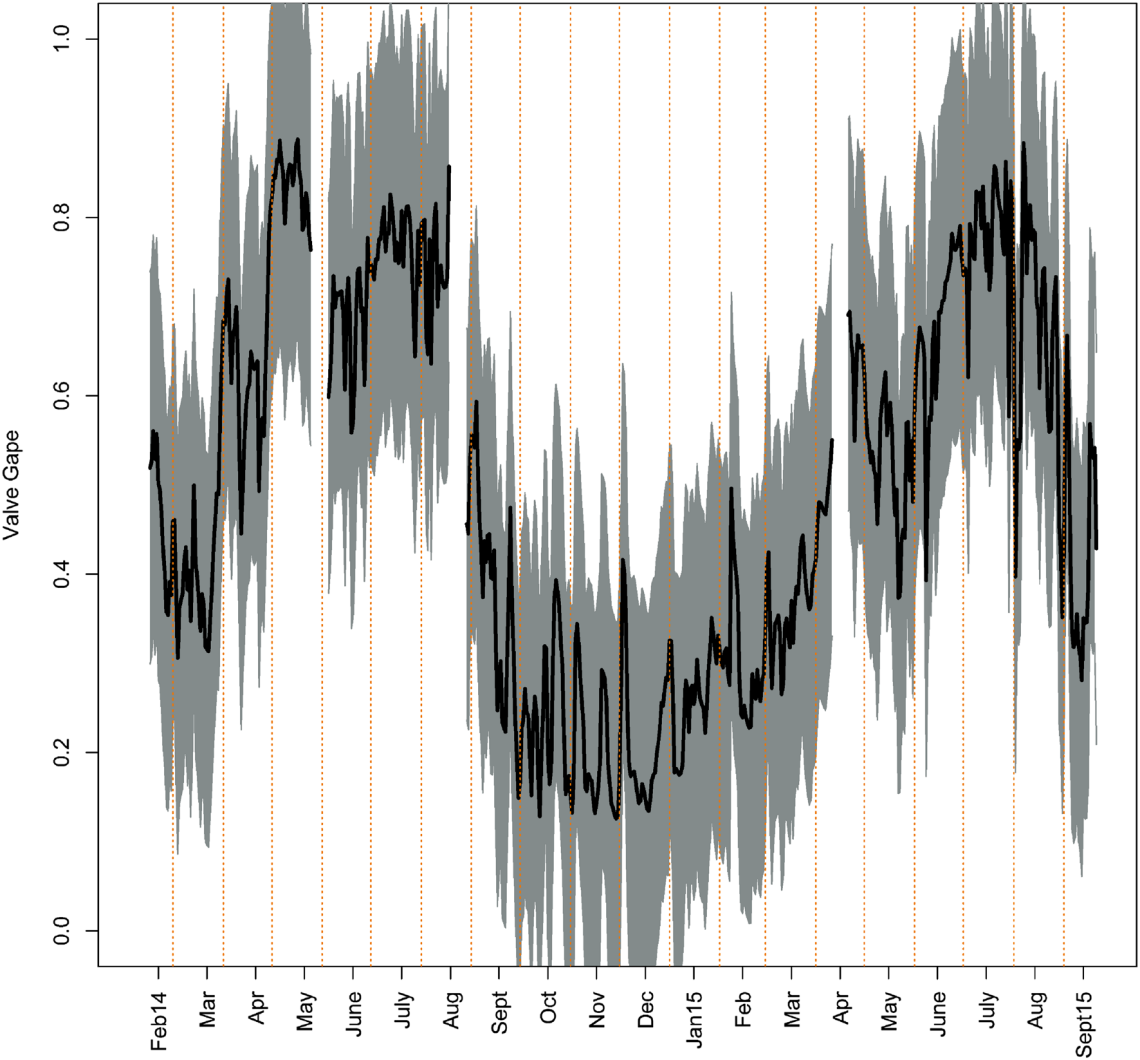
Valve gape daily mean (*black line*) and SD (*gray shadow*) by month from all available specimens per unit of time (*n* = 7–15)

* Specimen that died (B665). B677–B684 (*n* = 6) were the smaller replacements collected later in the experimental period (Figs. 2, 3 Online Resource 1). Gaps indicate that the channel malfunctioned during that period

### Long-term environmental measurements

Self-logging sensors for temperature (°C), salinity (PSU: Practical salinity unit), turbidity (FTU: Formazine Turbidity Unit), [Chl-a] (µg L^−1^), and light conditions (PAR: Photosynthetic Available Radiation measured as µmol m^−2^ s^−1^), were mounted on the lander over the entire deployment period.

Temperature and salinity were measured by a DST CT system (STAR-ODDI Data Storage Tag Conductivity and Temperature logger; http://www.star-oddi.com/) attached to the upper triangle part of the lander, 90 cm above the seabed. The CT system recorded temperature and salinity every 30 min.

Turbidity, [Chl-a], and light conditions were measured using two versions of JFE ocean instruments (http://ocean.jfe-advantech.co.jp). Wipers cleaned the sensor surface every 30 min immediately before a burst of 10 measurements. Light conditions were recorded with an upwards facing COMPACT-LW-ALW-CMP. Turbidity and [Chl-a] were measured with an Infinity-CLW-ACLW2-USB which was oriented parallel to the seabed 90 cm above the bottom.

Sea level records (in cm; reference Lowest Astronomical Tide) from the nearest tidal station (Hammerfest; http://www.kartverket.no/sehavniva/) were examined for the possible influences of tides on valve gape activity.

### Experimental specimens

In February 2014, adult specimens of *A. islandica* were collected with a 30-cm clam dredge in Sanden Bay, Ingøya (Fig. 1 Online Resource 1). Sixteen specimens were selected for the valve gape monitoring experiment. Another 12 specimens were collected and placed in additional cups on the lander next to the valve gape monitors for future studies (Fig. 1). Shell heights were 63.7–78.5 mm (±0.1 mm) at the beginning of the experiment. Each specimen was labeled with a shellfish tag (Glue-On shellfish tags FPN 8 × 4 mm; http://www.hallprint.com/). For this, a small portion of the periostracum in the umbonal region was abraded away, after which the label was adhered with cyanoacrylate glue.

During the entire experimental period, a total of 21 individuals were used (Table 1). One specimen died (*A. islandica* B665) and seven of the larger specimens were replaced with smaller ones (SH 50.7–56.6 mm) collected from the same population. Some of these individuals were used in continuing growth studies.

The lander was deployed for the first time on 7 February 2014 using a traditional coastal Norwegian fishing boat (sjark) “Fjord Strup”. Since this first deployment, the benthic lander was retrieved for maintenance and data collection twice per year (see Table 1). In the laboratory, all shells were measured, the data were downloaded from all instruments, and the instruments were cleaned, serviced and reprogrammed. Before redeployment, the aluminum lander frame was cleaned to remove overgrowth by fouling organisms. The time period from lander retrieval to redeployment was 1–3 days depending on weather conditions (Table 1). During these days, specimens were kept in their cups within 80 L baths of aerated sea water at ambient temperature, but were not fed.

### Statistical analysis

First, the synchrony among individuals was tested by calculating the pairwise correlation factor between all individuals and then between each of them and the average valve gape d^−1^ of all specimens [${\text{mean }}(G_{t 1 - n} )$]. The number of individuals ($n$) per time ($t$) varied among periods due to technical problems in some recorder channels (Table 1).

Second, we applied a standard multiple regression to identify which environmental factors were related to the average valve gape of the specimens in the experimental setup. A logit transformation of the average valve gape d^−1^ (${\text{logit[mean}}(G_{t 1 - n} )]$) was applied to fulfill linear modeling assumptions (Warton and Hui 2011). To avoid collinearity, explanatory variables were included in the analyses only when they had a (Pearson) correlation coefficient ≤±0.5 (Graham 2003; Duncan 2011; Ieno and Zuur 2015).

Third, an alternative approach was employed to address possible collinearity among the explanatory variables. PCA (Principal Component Analysis) was conducted on the environmental explanatory variables and the scores of the principal components were used as new explanatory variables in a subsequent multiple regression analysis (Graham 2003). This method allowed us to include all the original variables in the regression model, avoiding the subjective process of variable dropping (Graham 2003).

Finally, the two statistical approaches were compared. All analyses were done in R version 3.2.2 (www.r-project.org) and PAST3 (http://folk.uio.no/ohammer/past/).

## Results

### Environmental records

#### Sea level

Sea level variation was ±0.5 m for all the experimental periods, except one day in May 2014 when a single 1-m variation occurred (Fig. 2a). This indicates that storms have an additional effect on sea level in the bay beyond the tidal influence (http://www.kartverket.no/sehavniva/).

### Temperature

The daily average water temperature in Sanden Bay over the deployment period was 2.4–10.1 °C (Fig. 2b). In both 2014 and 2015, August was the warmest month with an average monthly temperature of 9.0 °C. In 2014, the coldest month was March with an average temperature of 3.7 °C, while in 2015 the coldest month was February with an average temperature of 3.3 °C. There were, however, individual measurements with recorded temperatures <1.6 °C in the first half of February 2015 and >10 °C in August 2014 and 2015. The lowest individual temperature record of 0.8 °C was recorded on February 3, 2015, and the maximum temperature of 10.3 °C occurred on August 9, 2014 and August 27, 2015.

### Salinity

The daily average salinity range was 30.8–34.4 (Fig. 2c). The minimum average daily value (30.8) was observed on February 10, 2015. However, there were occasional values <30 between January 28 and February 16, 2015. A large, sustained decrease in salinity occurred in both summers following the spring melt. In the summer of 2015, salinity decreased from 33.4 on June 21 to 32.3 on September 21 (Fig. 2c). We observed not only a seasonal change in salinity but also a gradual decrease over the entire experimental period.

### Light

Because Ingøya is ~500 km north of Arctic Circle, there are immense seasonal changes in light. Light levels rapidly decrease in October and November, while in December and early January there is no appreciable daylight at all. Light levels increase again in late January and February (Fig. 2d). The rapidly increasing light levels in February are accompanied by high levels of [Chl-a] in Sanden Bay by mid-March (Fig. 2d, e), when sea temperatures still are at or near their lowest for the year (Fig. 2b). From mid-May to mid-July the sun does not set, providing perpetual daylight. In May 2014, however, we recorded an artificial light reduction due to algal overgrowth on the JFE sensor screen (Fig. 2d).

### [Chl-a] and turbidity

Turbidity ([FTU]) values were measured by optical backscatter (OBS) to estimate water transparency, and [Chl-a] (µg L^−1^) by fluorescence as an indication of primary productivity in Sanden Bay. There were well defined, but episodic [Chl-a] peaks in mid-February 2014 and in mid-January in 2015 (Fig. 2e). These transient peaks during winter, each lasting only 1–2 days, were likely due to the effect of winter storms leading to sediment resuspension releasing buried [Chl-a]. This is supported by turbidity values, which also showed transient spikes in February 2014 and January 2015, with maximum values of 44 and 48 FTU, respectively (Fig. 2f). More sustained [Chl-a] peaks occurred during spring and summer, reaching the highest values in May and August, respectively (Fig. 2e). In 2014, [Chl-a] varied from 0.1 to 348.1 µg L^−1^. The extremely high values measured in spring were due to algal overgrowth on the JFE sensor screen. In 2015, the sensor problem was solved by covering the sensor with a PVC cylinder to exclude light and therefore eliminate the algae on the sensor. The [Chl-a] in 2015 were 0.1–57.1 µg L^−1^.

### Valve gape monitors

There were significant differences in monthly valve gape activity (*P* < 0.01; one-way ANOVA), with monthly means of 0.19–0.84 (19–84% of the valve gape total magnitude, respectively; Fig. 3). Valve gape measurements over the two calendar years showed a well-defined activity cycle in *A. islandica* (Fig. 3). There were distinct types of gaping activity levels that were consistent between years as well as among individuals. We discerned two levels of activity, i.e., “active” with an average valve gape >0.2, and “inactive” with an average valve gape ≤0.2 (Fig. 3 and Figs. 2, 3 Online Resource 1). Values <~20% represent >95% probability of valves being closed in bivalves (Jou et al. 2013).

**Fig. 4:**
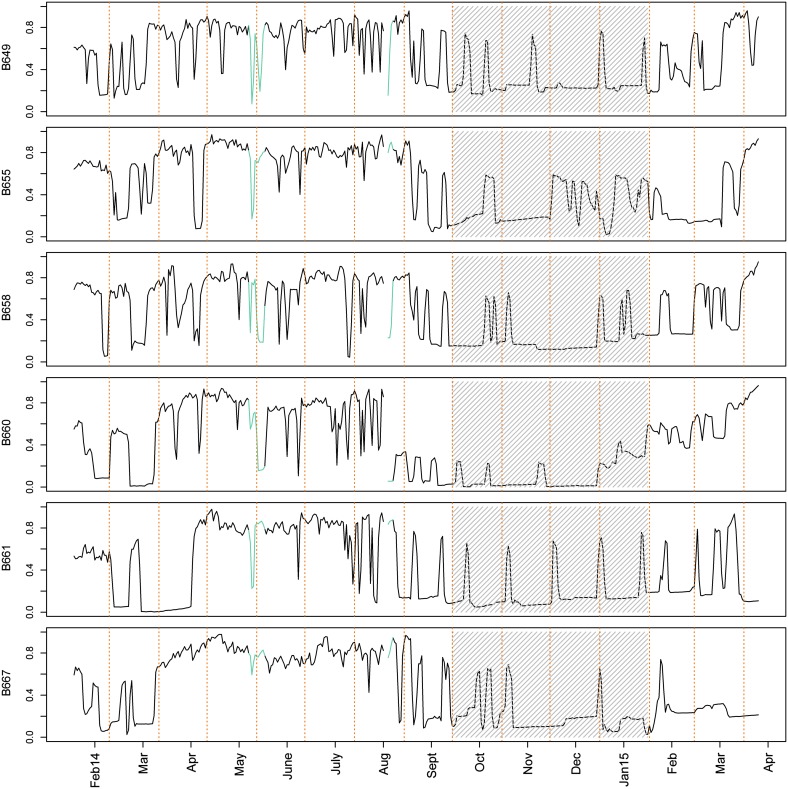
Gaping activity of six *A. islandica* specimens (Table 1) February 2014–April 2015. *Green line* highlights a week of valve gape data after the lander was deployed (excluded from analyses; May and August). *Gray hashed* background highlights common inactive period for all the specimens (average valve gape <0.2), which includes periodic gaping lasting 1–3 days

On average, the sample population of *A. islandica* individuals was inactive from the beginning of October to the end of January (with an average daily gape <23%; Fig. 3). That inactivity period, however, started earlier (mid-September) and/or finished later (end February/early March) in some specimens (Figs. 4 and Figs. 2, 3 Online Resource 1). During that time, they opened widely once or twice a month for 1–3 days (Fig. 4 and Figs. 2, 3 Online Resource 1). Furthermore, we observed that all specimens required around a week to recover their normal activity after lander redeployment (Fig. 4, green line). Consequently, those data were not considered in the statistical analysis.

In both 2014 and 2015, a consistent transition from low valve gape to high started between February and March. The daily mean valve gape then increased considerably between the months of March and April, from 0.41 to 0.63 in 2014, and from 0.36 to 0.56 in 2015 (Fig. 3). During summer 2014 (June 21–September 21), the daily average valve gape was 0.65 and ranged from a maximum of 0.86 in mid-August to 0.22 in September. In summer 2015 the results were quite similar, with a daily average valve gape of 0.66, ranging from 0.88 in early August to 0.28 in September. During both years, the highest, continuous level of activity occurred in late spring to early summer. Valve gape monthly means reached their maximum in May 2014 (0.84) and in July 2015 (0.78; Fig. 3).

### Valve gape activity vs. environmental records

**Fig. 5:**
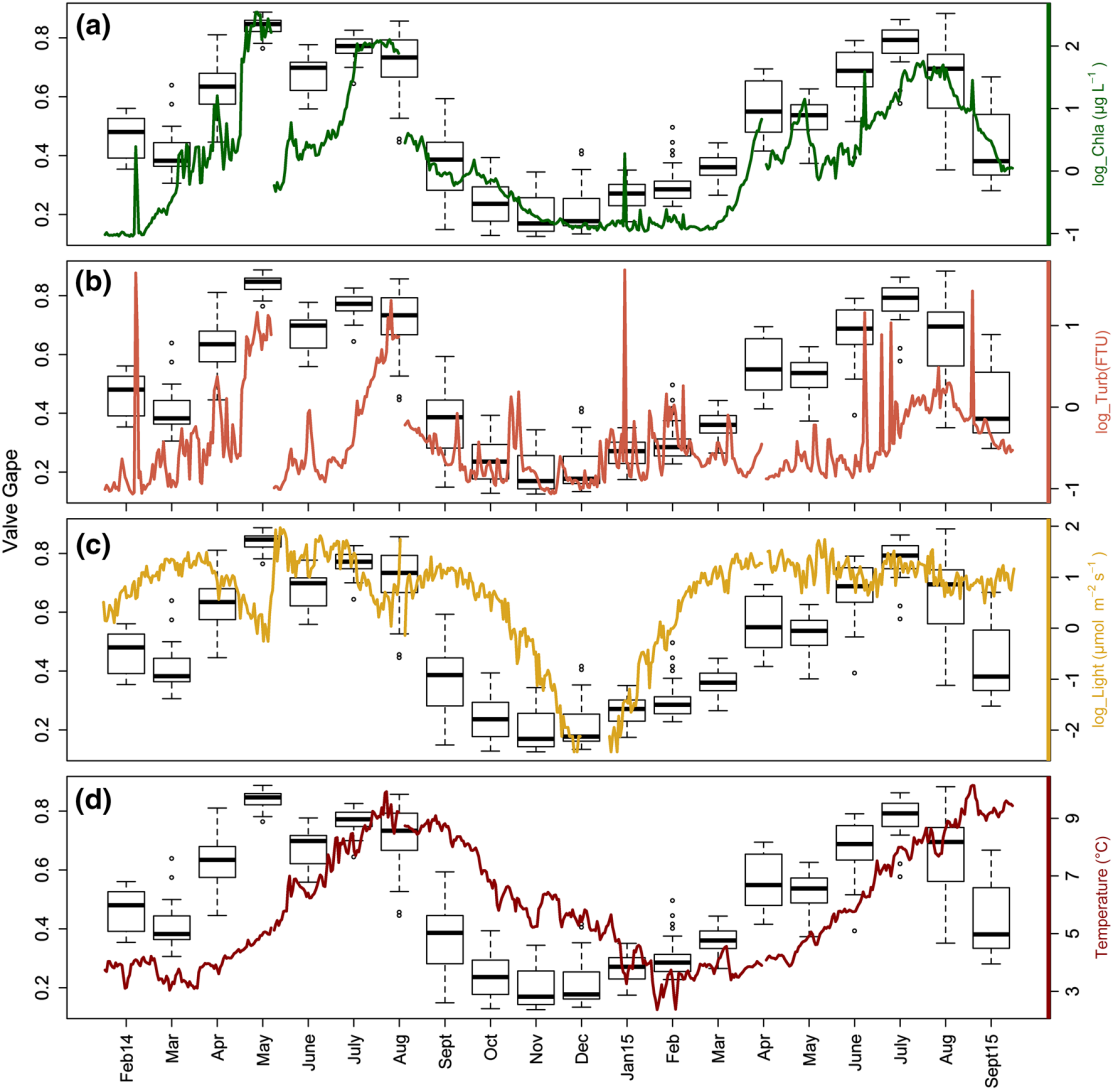
*Boxplox* per month of daily average valve gape of all the specimens vs. key environmental variables: **a** [Chl-a] **b** Turbidity, **c** Light, **d** Temperature

When average valve gape activity was compared with daily means of the different environmental variables, [Chl-a] was the variable with the highest correlation (*r* = 0.8; *P* value < 0.01; Fig. 5a), followed by turbidity (Fig. 5b), sea level, light (Fig. 5c), temperature (Fig. 5d), and salinity (Table 2). The periods with the highest valve gape coincided with the highest levels of [Chl-a] (Fig. 5a). It is also apparent that the seasonal change in valve gape activity was temporally offset from temperature, with higher valve gape values leading the temperature pattern by 2–3 months (Fig. 5d).

**Table 2:** Correlation between the daily average gaping activity of the specimens (AvgGape) versus the different environmental variables (left bottom)

	AvgGape	Temperature	Salinity	Light	Sea level	Log_[Chla]	Log_Turb
AvgGape		0.00	0.01	0.00	0.00	0.00	0.00
Temperature	0.30		0.00	0.08	0.00	0.00	0.00
Salinity	0.12	−0.25		0.00	0.05	0.30	0.09
Light	0.41	0.08	0.18		0.00	0.00	0.00
Sea level	−0.48	−0.24	−0.08	−0.28		0.00	0.00
log_[Chla]	**0.82**	0.53	−0.04	0.20	−0.47		0.00
log_Turb	0.50	0.28	−0.07	−0.18	−0.25	0.71	

The highest correlation with AvgGape is shown in bold

The top right shows the *P* values of the corresponding correlations

Turbidity was highly correlated with [Chl-a] values (*r* = 0.71; *P* value < 0.01). When primary production increased in the bay, water transparency was reduced (Fig. 2d, e). Because of the correlation with [Chl-a], this variable was removed from the standard multiple regression analysis but included in the PCA regression.

Sea level had a negative relationship with gaping activity due to its influence on other environmental factors (Table 2). When sea level increased, there was less [Chl-a] available (*r* = −0.47; *P* value < 0.01) and less light at the sea bottom (*r* = −0.28; *P* value < 0.01). An increase in sea level was furthermore associated with a decrease in water temperature (*r* = −0.24; *P* value < 0.01) and salinity(*r* = −0.08; *P* value < 0.01). This illustrates the influence of tides and storms on water exchange within Sanden Bay and seems to indicate that local primary production drives the bay’s productivity.

### Synchrony among individuals

**Fig. 6:**
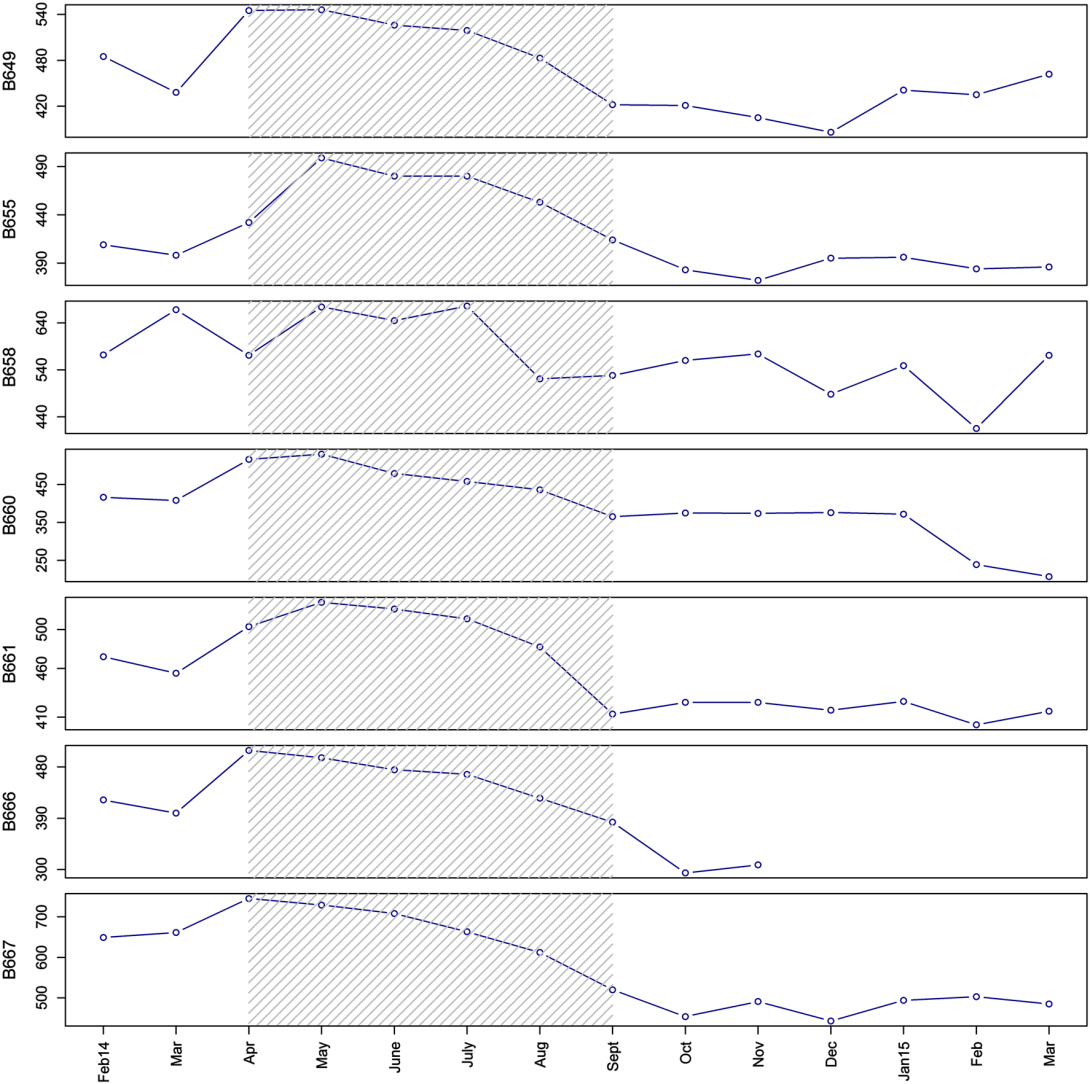
Maximum signal per month of *A. islandica* specimens from recorder 1 (except specimen B665 which died). *Gray dashed* background highlights months with higher average gape valve (>0.5). There are different scales on the *y*-axis to better illustrate the change in the monthly maximum signal per specimen. Recorder 2 specimens are not shown because R2 was adjusted in August 2014, so raw data were not comparable

There was high synchrony in daily gaping activity among all specimens. Further, there was also high synchrony between individuals and the average gape of all specimens (Fig. 3 and Figs. 4, 5, 6 Online Resource 1). Correlation factors ranged 0.5–0.9. Specimen B665 was the only one which died (end of August 2014), and which correlated poorly with the rest of the population (Fig. 4 Online Resource 1).

### Multiple regression

After linearity exploration of the environmental variables with the dependent variable, we log-transformed the variables [Chl-a] and Turbidity. Turbidity was removed from the analysis due to high correlation with [Chl-a] (*r* = 0.7; Fig. 7 Online Resources 1). A stepwise variable selection procedure (both directions) did not lead to exclusion of any other measured explanatory variables. Consequently, we modeled the logit of the average daily valve gape with a dependency on Temperature, Salinity, log [Chl-a], Light, and Sea level:

$${\mathbf{M1}}: y_{i} = \beta_{0} + \beta_{1} \times {\text{Temperature}}_{i} + \beta_{2} \times {\text{Salinity}}_{i} + \beta_{3} \times { \log }\_[{\text{Chla}}_{i} ] + \beta_{4} \times {\text{Light}}_{i} + \beta_{5} \times {\text{Sea level}}_{i} + \varepsilon_{i} ,$$

where $y_{i}$ was the logit of the average valve gape on day $i$ ($i$ range 1–592), and $\varepsilon_{i}$ the model error on day $i$ ($\varepsilon_{i\sim } N\left( {0,1} \right)$).

Model M1 explained 75% of the variation in the average daily valve gape of *A. islandica* (adjusted *R* ^2^ = 0.75, *F*_1,66_ = 328; Table 3). All the variables were statistically significant (*P* value < 0.05). Residual plots showed a good fit of the model (Fig. 8 Online Resources 1). We then explored the contribution of each variable to the total model (M1.1–M1.4; Table 1 Online Resources 1). [Chl-a] was the main contributor to M1, individually explaining 66% of the gaping activity variance. When the variable light was added to the single variable [Chl-a] model, the two variables explained 72% of the variability in valve gape through time. This is roughly equivalent to the full M1 model (75%; Table 1 Online Resources 1). Thus [Chl-a] and light were the most important explanatory variables for the seasonal cycle in valve gape.

**Table 3:** Regression table for model M1

M1 logit(AvgGape)	Coefficient	Std. error	*T* value	*P* value
(Intercept)	−4.041	1.793	−2.253	0.0246
Salinity	0.143	0.052	2.760	0.0059
Temperature	−0.088	0.013	−6.720	4.63E−11
Light	0.021	0.002	9.745	<2E−16
Sea level	−0.004	0.002	−2.335	0.0199
log_[Chl-a]	0.903	0.030	29.942	<2E−16
			*R* ^2^-adjusted = 0.75	

### PCA regression

First, a PCA was conducted on the observations of the explanatory variables to extract the common signal from all of them (PCA; Table 4). This approach prevents the loss of explanatory power resulting from exclusion of variables (Carnes and Slade 1988; James and McCulloch 1990). PC1 accounted for 38.7% of the variability among the variables (*λ* = 2.32), with log [Chl-a] driving the loadings positively (with a correlation with PC1 = 0.6; Fig. 6; Table 4). PC2 explained 22.8% of the remaining variability among the variables (*λ* = 1.37), and was best represented by light conditions (correlation with PC2 = 0.7; Fig. 6; Table 4). See Table 4 for principal components with Eigenvalue <1.

**Table 4:** Correlation table between key variables and principal components (top) and multiple regression model M2 summary (bottom)

Variance explained	PC1 (38.7%)	PC2 (22.8%)	PC3 (16.3%)	PC4 (10.4%)	PC5 (9.1%)	PC6 (2.7%)
Temperature	0.45	−0.17	−0.43	**0.48**	−**0.55**	0.23
Salinity	−0.09	**0.57**	**0.61**	0.41	−0.35	0.02
Light	0.13	**0.66**	−**0.44**	0.18	0.50	0.25
Sea level	−0.41	−0.37	0.01	**0.74**	**0.38**	−0.09
log_[Chl-a]	**0.61**	0.02	0.11	0.15	0.22	−**0.74**
log_Turbidity	**0.49**	−0.26	0.49	0.02	0.36	**0.57**
M2 (*R* ^2^-adjusted = 0.75)	x***	x***	x***	x*	x***	x***

Numbers in bold indicate the two main environmental factors driving the principal component (PC)

x*** = significant variable with *P* value = 0, x** = significant variable with *P* value < 0.01, x* = significant variable with *P* value < 0.05 and, *x* = not significant variable

Ultimately, multiple regression analysis was conducted using the scores of the principal components as explanatory variables and the logit of average daily valve gape as response variable (Graham 2003):

$${\mathbf{M2}}: y_{i} = \beta_{0} + \beta_{1} \times {\text{PC}}1_{i} + \beta_{2} \times {\text{PC}}2_{i} + \beta_{3} \times {\text{PC}}3_{i} + \beta_{4} {\text{PC}}4_{i} + \beta_{5} \times {\text{PC}}5_{i} + \beta_{5} \times {\text{PC}}6_{i} + \varepsilon_{i} ,$$

where $y_{i}$ was the logit of the average valve gape on day $i$ ($i$ range 1–592), and $\varepsilon_{i}$ the model error on day $i$ ($\varepsilon_{i\sim } N\left( {0,1} \right)$). This regression model (M2) explained 75% of the valve gape variance (adjusted *R* ^2^ = 0.75, *F*_1,66_ = 273; Table 4 and Table 2 Online Resources 1), with significant values for all the variables (*P* value < 0.05; Table 4). Thus, the M2 results were identical to those yielded by M1, where 75% of the gaping activity variance was also explained. Therefore, excluding turbidity from M1, did not influence our results.

In summary, the standard multiple regression results (M1) were supported by the PCA regression model (M2). These two statistical approaches and the consistency in the results from them, clearly suggest that [Chl-a], followed by light conditions, are the main environmental drivers of *A. islandica* gaping activity. While other variables may have some relevance and have a relationship at a particular time, these were generally far less influential in relation to the valve gape of this northern Norwegian population (Table 1 Online Resources 1).

## Discussion

Our field experiment addresses the critical need to study *A. islandica* biological activity at several temporal scales. We documented the in situ daily and seasonal gaping activity of this bivalve in relation to environmental factors that drive their rhythms. Valve gape in *A. islandica* exhibits a well-defined seasonal pattern which is mainly driven by [Chl-a].

Although the experimental organisms had electro-coils attached to the outside of their shells and were each kept in individual cups, there is no indication that this experimental setup impacted the study results or considerably modified the valve gape behavior. Over the study period only one specimen died and we furthermore observed that the specimens buried themselves deep into the cups. Moreover, an analysis of the body mass index (BMI = Dry Weight-Ash Weight/Height^3) showed that individuals from the experiment had the same or even slightly higher BMI than individuals freshly collected from the field at the time the lander was recovered. Results from other species show that electro-coils attached to the external shell surface had no influence on their behavior (Tran et al. 2011; Jou et al. 2013).

### Valve gape vs. growth

Valve gape activity in bivalves is related to important physiological processes including feeding and respiration (Bayne 1998; Markich 2003; Riisgård et al. 2003; García-March et al. 2008). Filter-feeding bivalves open their valves to extend their siphons and filter the surrounding water. Witbaard et al. (1997b) demonstrated in a laboratory experiment that a large proportion of the inter-specimen variation in shell growth of *A. islandica* could be explained by differences in individual feeding activities. This suggests a link between valve gape, open siphons, and shell growth, i.e., that valve gape is indicative of shell growth. Unfortunately, we were unable to collect small specimens at the start of the experiment which might have enabled a posteriori determination of this relationship. The specimens used were too large and too slow-growing to measure shell growth with calipers, given the measurement error (Thompson et al. 1980). We used an alternative method to determine whether the seasons with wide open valves (≥50% gape April–September) and closed valves (≤20% October–end of January) coincided with periods of shell growth and non-growth. These thresholds (20 and 50%) are based on Jou et al. (2013), representing >95% probability of valves being closed or, alternately, siphons extended, respectively, in *Corbicula fluminea*. We used the change in monthly maximum (raw) valve gape signal, i.e., the minimum measured distance of completely closed valves. A progressively decreasing signal strength (or drift) indicated that the valves and the electro-coils became progressively further apart when closed. The growing shell margin pushes the sensors away from each other, directly indicating shell growth (Schwartzmann et al. 2011; Massabuau et al. 2015). Six out of seven specimens exhibited a strong trend of decreasing signal (maximal) strength between April and September 2014 (Fig. 6) followed by a more stationary period without a clear trend in the maximum signal. These periods coincided with the periods of high and low valve gape values, respectively. This suggests that periods with high valve gape activity and high [Chl-a] corresponded to a progressive decline in maximum signal strength (increase in minimum valve gape) and therefore shell growth. These results are supported by earlier studies that found the highest shell growth rates for this species in spring and early summer (Thompson et al. 1980; Schöne et al. 2005; Witbaard and Hippler 2009), and provide a link between food availability ([Chl-a]), high valve gape activity, and shell growth.

The valve gape data presented here, thus, suggest an ‘active’ growth season of about eight months for this location in northern Norway. Some studies have previously correlated their *A. islandica* growth chronologies with Sea Surface Temperature (SST) or salinity data from February to September (northeast Iceland; Marali and Schöne 2015). Our study clarifies the ecological and biological basis for using this period.

In contrast to our findings, Mette et al. (2016) described a growing season of 12 months from the same population in Sanden Bay (from April to March/April of the following year). Their results, however, were based on shell oxygen isotopes values from only two subsampled annual increments. Our results are based on daily averages of gaping activity from a minimum of 7 simultaneously measured specimens. Although oxygen isotopes are excellent tools for reconstructing long time series of environmental annual variability, more replication (of individuals and years) and/or fine scaler sampling of the shell increments is needed when addressing sub-annual resolution (DeLong et al. 2013; Schöne and Gillikin 2013).

### Valve gape vs. environment

Average daily valve gape was highly synchronized (*r*_all animals_ >0.5) and showed clear seasonal differences. High synchrony in valve gape has also been observed in other bivalves such as *Mya arenaria*, *Dreissena polymorpha*, *Crassostrea gigas*, and *Pinna nobilis* suggesting that a common external force with a periodicity similar to the activity drives such a response (Thorin 2000; Borcherding 2006; Mat et al. 2012; García-March et al. 2016).

We measured various environmental variables of which [Chl-a], light, and temperature are the most likely to explain the seasonal pattern in shell gape. *Arctica islandica* is a poikilotherm and its activity and growth is directly dependent on ambient temperature (Winter 1969; Clarke 2003; Hiebenthal et al. 2012). There are, however, conflicting results on the significance of temperature on *A. islandica* growth and gape activity. In laboratory growth experiments, faster growth at higher temperatures was reported (Witbaard et al. 1997b), with an added effect of salinity (Hiebenthal et al. 2012). Field studies were, however, not always conclusive about the role of temperature in shell growth. Some found significant correlation between SST and shell growth rate of *A. islandica* (Wanamaker et al. 2008; Butler et al. 2010; Marali and Schöne 2015), while others did not find such a strong relationship (Witbaard et al. 1996; Marchitto et al. 2000; Epplé et al. 2006; Stott et al. 2010). In a temperate environment, food and temperature are hard to separate as explanatory variables for activity or growth. The difficulty in disentangling these two variables in a field setting was one of the reasons we conducted this study at this northern location (71°N). Our results were consistent with other bivalve growth studies at high latitudes, which also found growth cessation at elevated temperatures coincident with low food availability (Carroll et al. 2009, 2011; Ambrose et al. 2012).

In this study, we observed that the population started to consistently open their valves and become active near the coldest period of the year (around March) and conversely closed their valves and started to become inactive by mid-September when temperatures were near their annual maximum (Fig. 5d). The valve gape records, however, show that they were not completely inactive in this winter period. All clams opened their valves widely once or twice a month for 1–3 days, and then closed again. A similar pattern has previously been observed in experiments and in the field (Taylor 1976; Strahl et al. 2011). While the reason for this behavior is not clear, it could be related to respiration and/or might be a type of probing behavior to test whether the environment is favorable. Our results showed that once there is food enough, the bivalves do not return to a dormant state but start feeding continuously with fully open valves. These results agree with earlier studies in *A. islandica* (Winter 1969) and other bivalves (Higgins 1980; Williams and Pilditch 1997; Riisgård et al. 2006) where the presence of Chl-a appears to be the main driver for sustained opening of their valves.

Next to [Chl-a], there was a relatively strong positive correlation between valve gape and light conditions. In some bivalve species valve gape behavior is directly triggered by light conditions (García-March et al. 2008; Schwartzmann et al. 2011), whereby variations in sun or moon irradiance immediately provoke a response. *Arctica islandica* is known to have a shadow reaction to light (Morton 2011), but our results did not indicate an immediate response of valve gape to moon phase, day length or hourly variations in light intensity (unpubl data). Light could have an indirect effect on *A. islandica* through modulation of food availability (Kaartvedt 2008). There is indeed evidence that for some species of bivalves, valve gape responds to the presence of algal food (Higgins 1980; Williams and Pilditch 1997; Riisgård et al. 2003, 2006. In laboratory conditions under continuous light exposure, *A. islandica* exhibited a 3–7 min periodicity in valve and mantle activity, which could be related to intrinsic drivers such as a biological clock (Rodland et al. 2006). The exact role of light as driving factor for valve gape of *A. islandica* remains unresolved, and the effect of photoperiod at different algal concentrations should be studied to clarify this issue.

In summary, our research found that: (1) gaping activity of *A. islandica* is highly synchronized among individuals in the studied population (2) [Chl-a] is the main driver of valve gaping activity in northern Norway, (3) the clams had a period of active gaping of eight months (between February and September). These results suggest the length of growing season in northern Norway is likely limited to about eight months (Weidman et al. 1994; Schöne et al. 2005; Dunca et al. 2009) starting very early in the spring and ending in late summer/early fall (Witbaard et al. 2003; Dunca et al. 2009).

## Electronic supplementary material

Below is the link to the electronic supplementary material.

Supplementary material 1 (PDF 644 kb)
