# Supplementary material for: Environmental factors regulating gaping activity of the bivalve Arctica islandica in Northern Norway
Source: Mar Biol. 2017 Apr 27;164(5):116. doi: 10.1007/s00227-017-3144-7 (PMC5409809; doi:10.1007/s00227-017-3144-7)
Supplement: Supplementary file 1 — Supplementary material 1 (PDF 644 kb) [file 227_2017_3144_MOESM1_ESM.pdf]

Online Resources 1

Journal: Marine Biology

Manuscript: "Environmental factors regulating gaping activity of the bivalve *Arctica islandica* in Northern Norway"

Ballesta-Artero, Irene[1]; Witbaard, Rob[2]; Carroll, Michael L.[3]; van der Meer, Jaap[1].

[1] NIOZ; Netherlands Institute for Sea Research and Utrecht University ,Department of Coastal Systems, PO Box 59, 1790 AB Den Burg, Texel, The Netherlands

[2] NIOZ; Netherlands Institute for Sea Research and Utrecht University, Department of Estuarine and Delta Systems, PO Box 140, 4400 AC Yerseke, The Netherlands

[3] Akvaplan-niva, FRAM-High North Centre for Climate and the Environment, 9296 Tromsø, Norway

Corresponding author: irene.ballesta.artero@nioz.nl / phone number: 0031 (0) 222 369 461

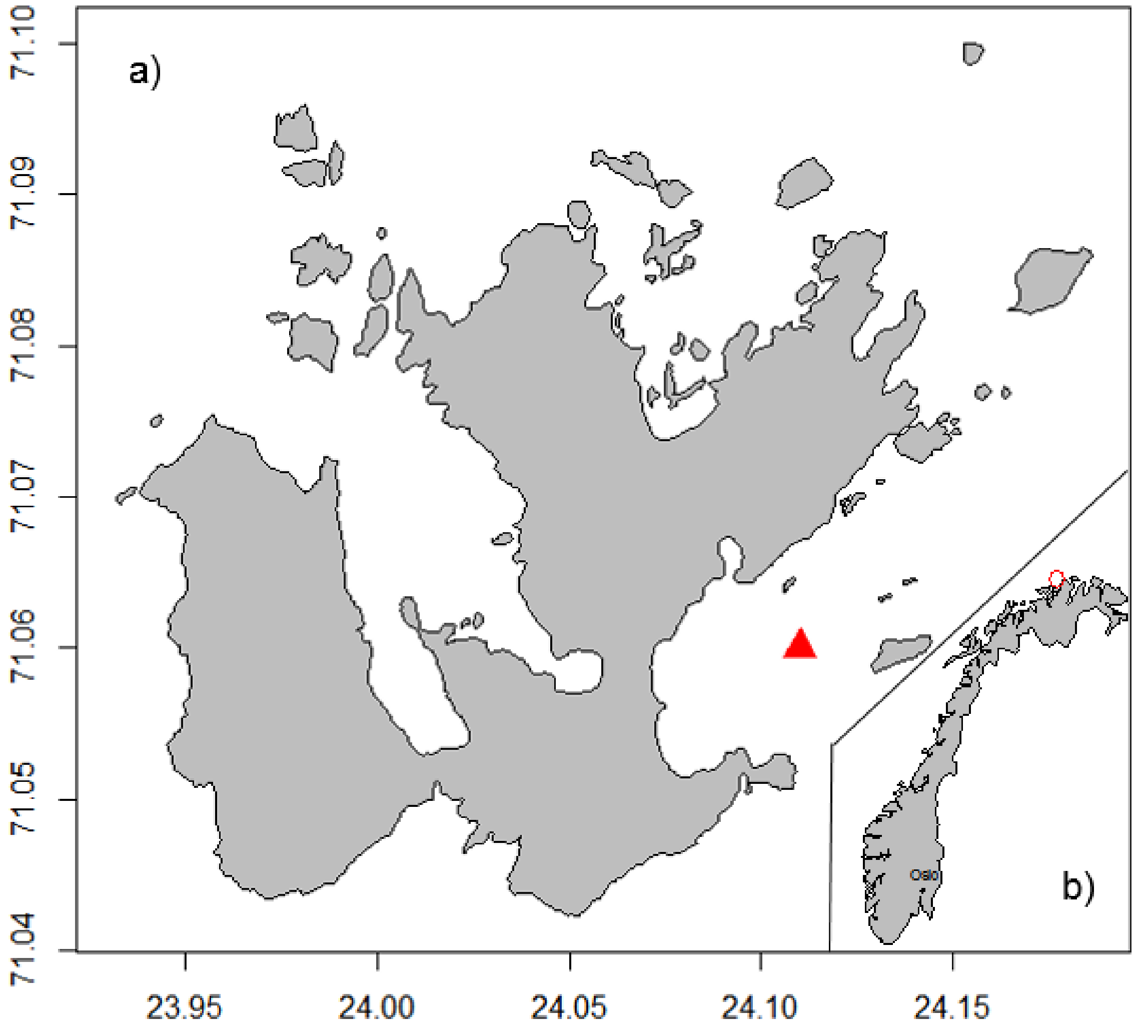

Fig. 1 a) Ingøya Island with triangle marking lander position, and b) Norway map with circle highlighting fieldwork location

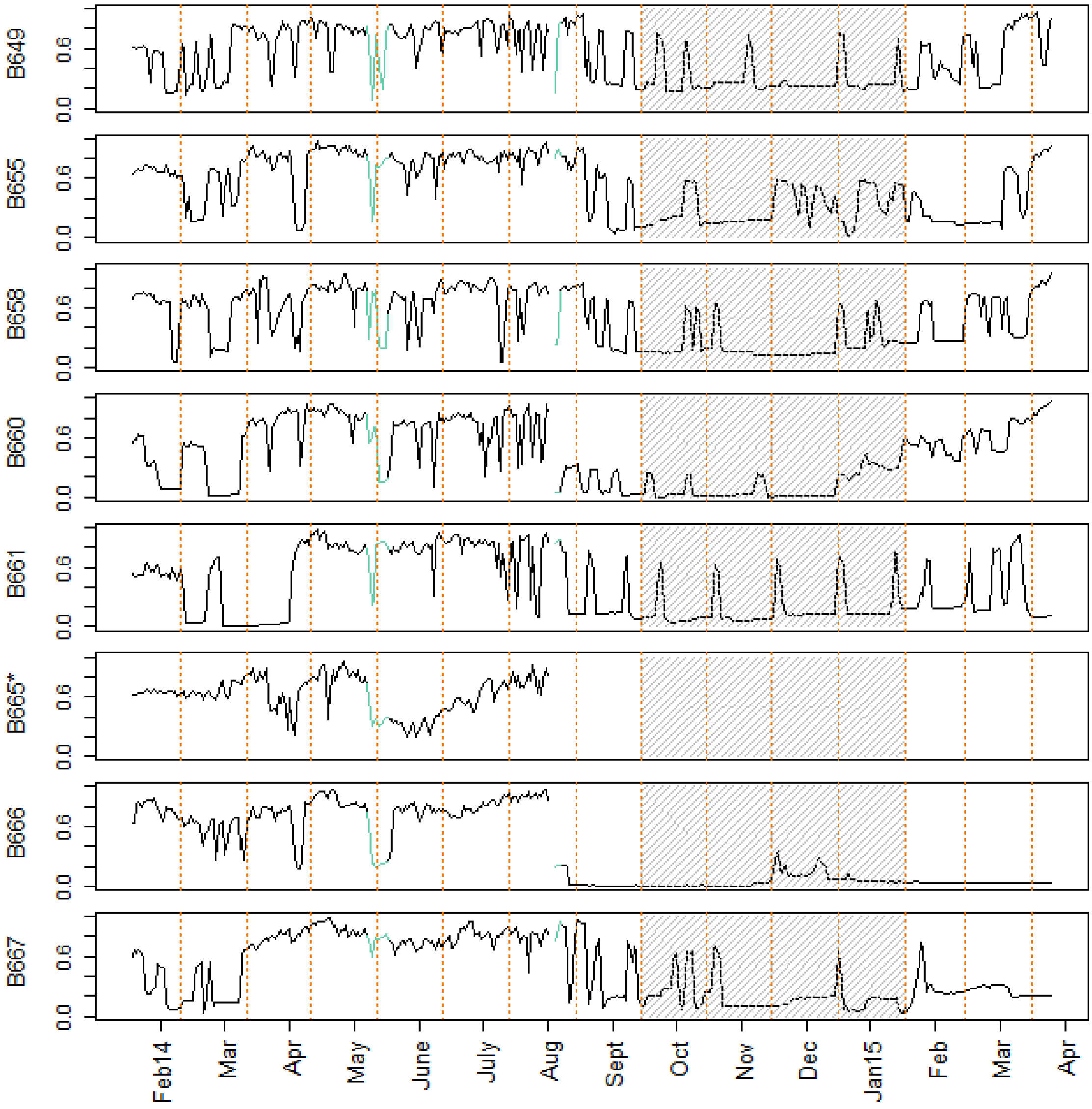

Fig. 2 All data from the eight *A. islandica* specimens in recorder 1. There was no data from April 2015 to September 2015 because the recorder flooded. B665* died at the end of August 2014. It was replaced by specimen B681, but there is no data because channel malfunctioned for that period. Grey hashed background highlights the common inactive period for all the specimens (average valve gape <0.2)

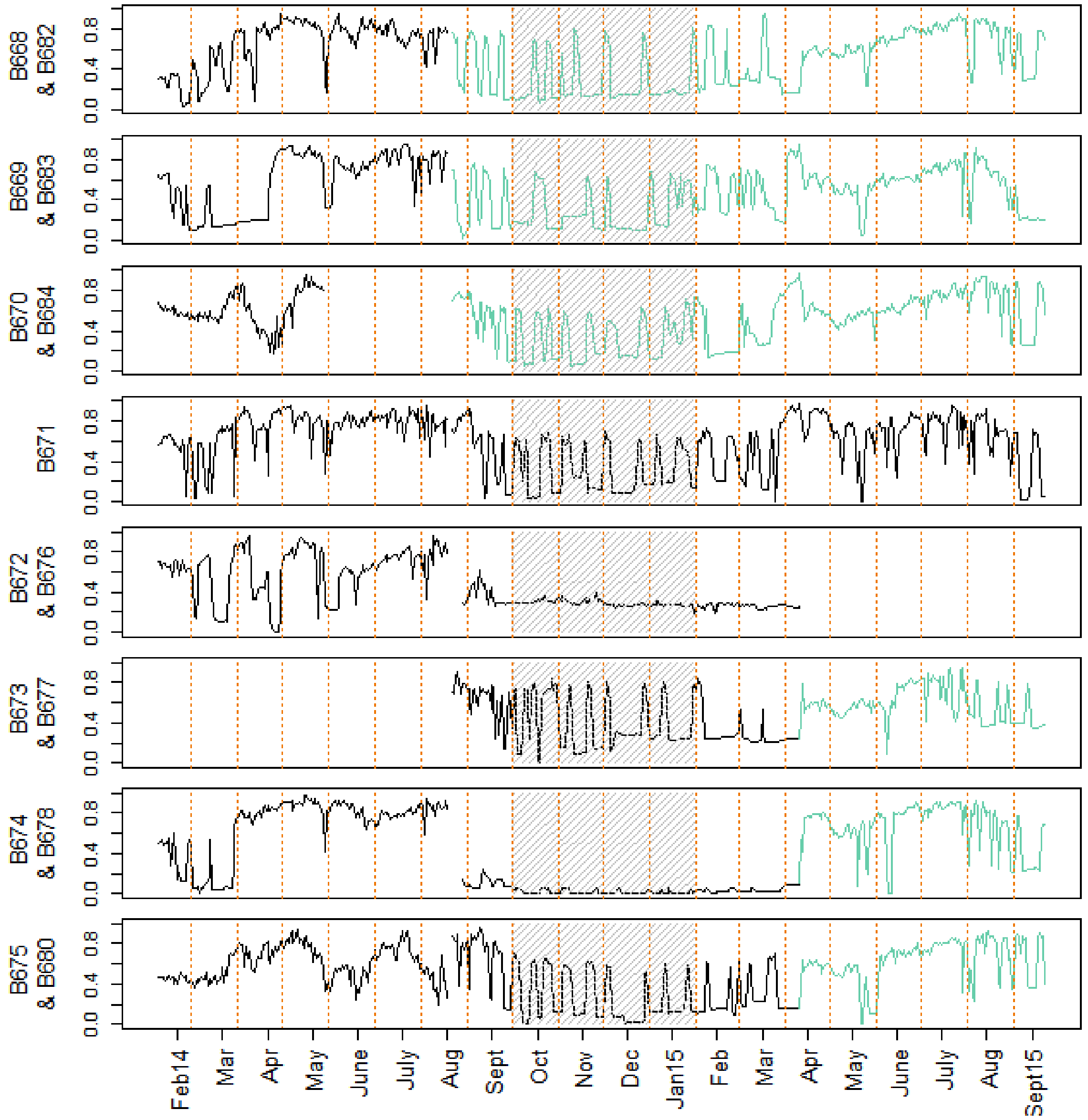

Fig. 3 All data from the 15 *A. islandica* specimens in recorder 2. Different line color indicates a change of specimen. No data for specimens B673 and B676 because the channels malfunctioned for that period. Grey hashed background highlights the common inactive period for all the specimens (average valve gape <0.2)

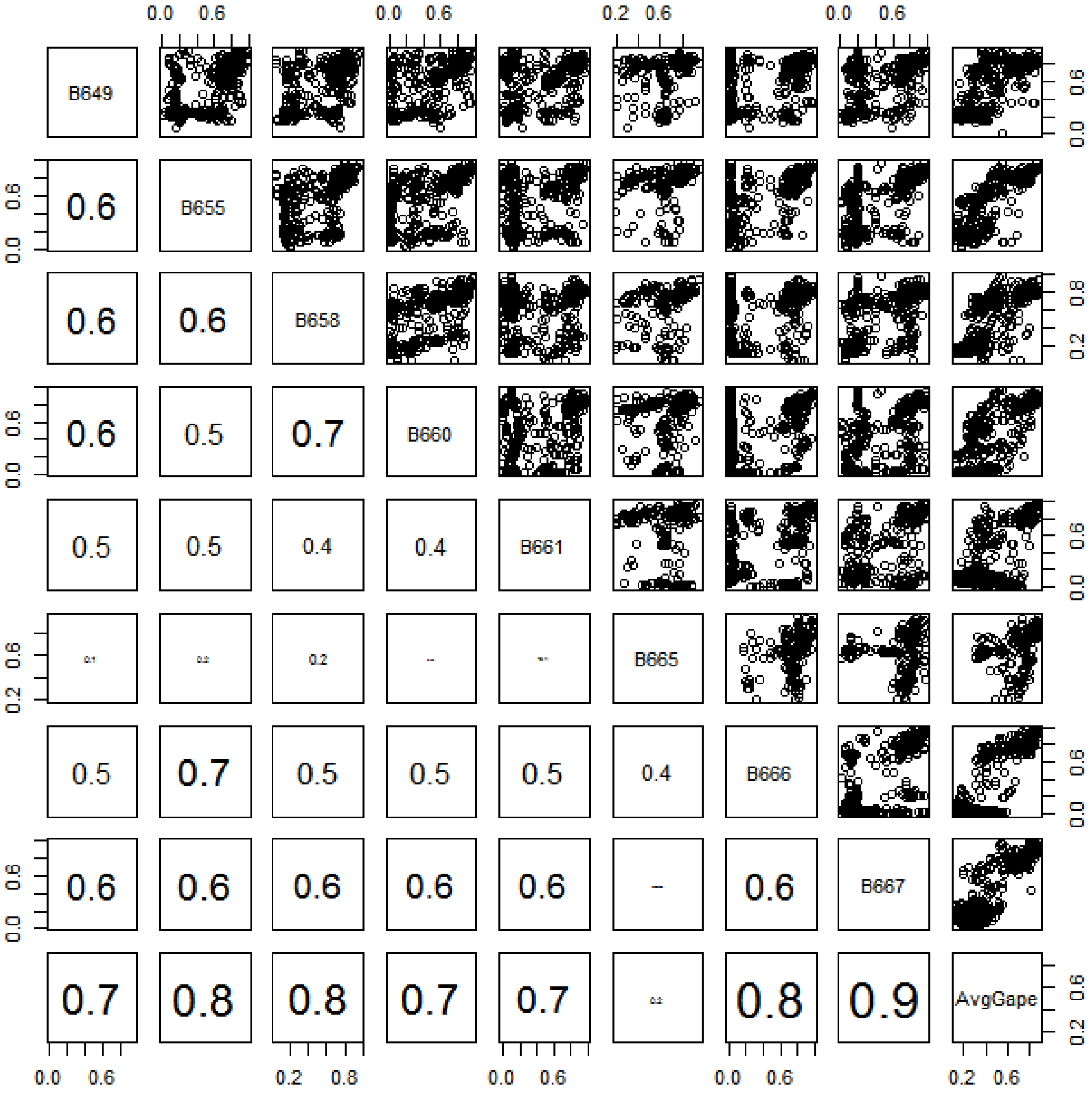

Fig. 4: Correlation plot between the original specimens from recorder 1 and the average valve gape of all the specimens (AvgGape). B665 died at the end of August 2014. No data for replacement specimen B681 because the channel malfunctioned for that period

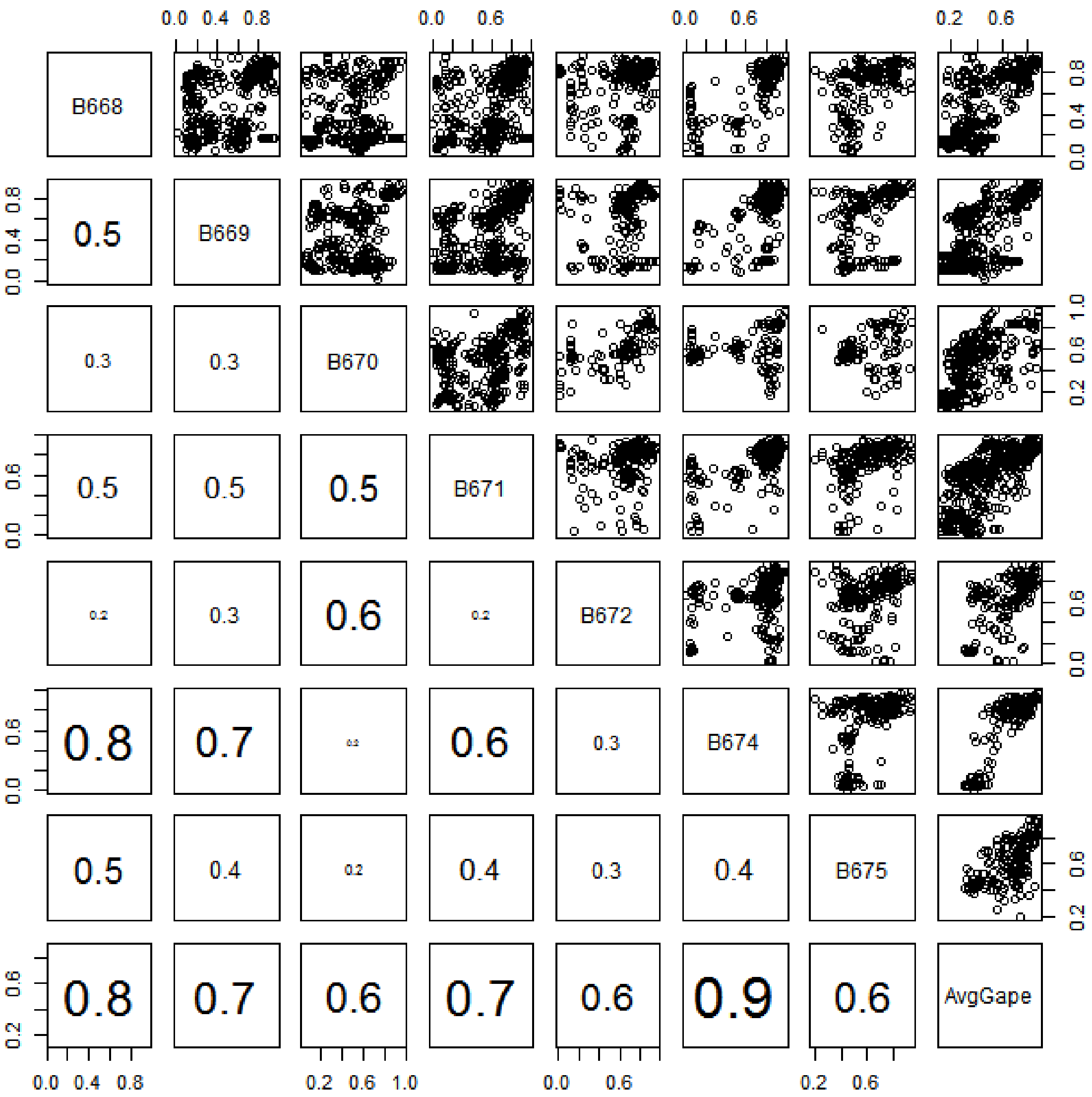

Fig. 5: Correlation plot between the original specimens from recorder 2 and AvgGape. No data for B673 because the channel malfunctioned

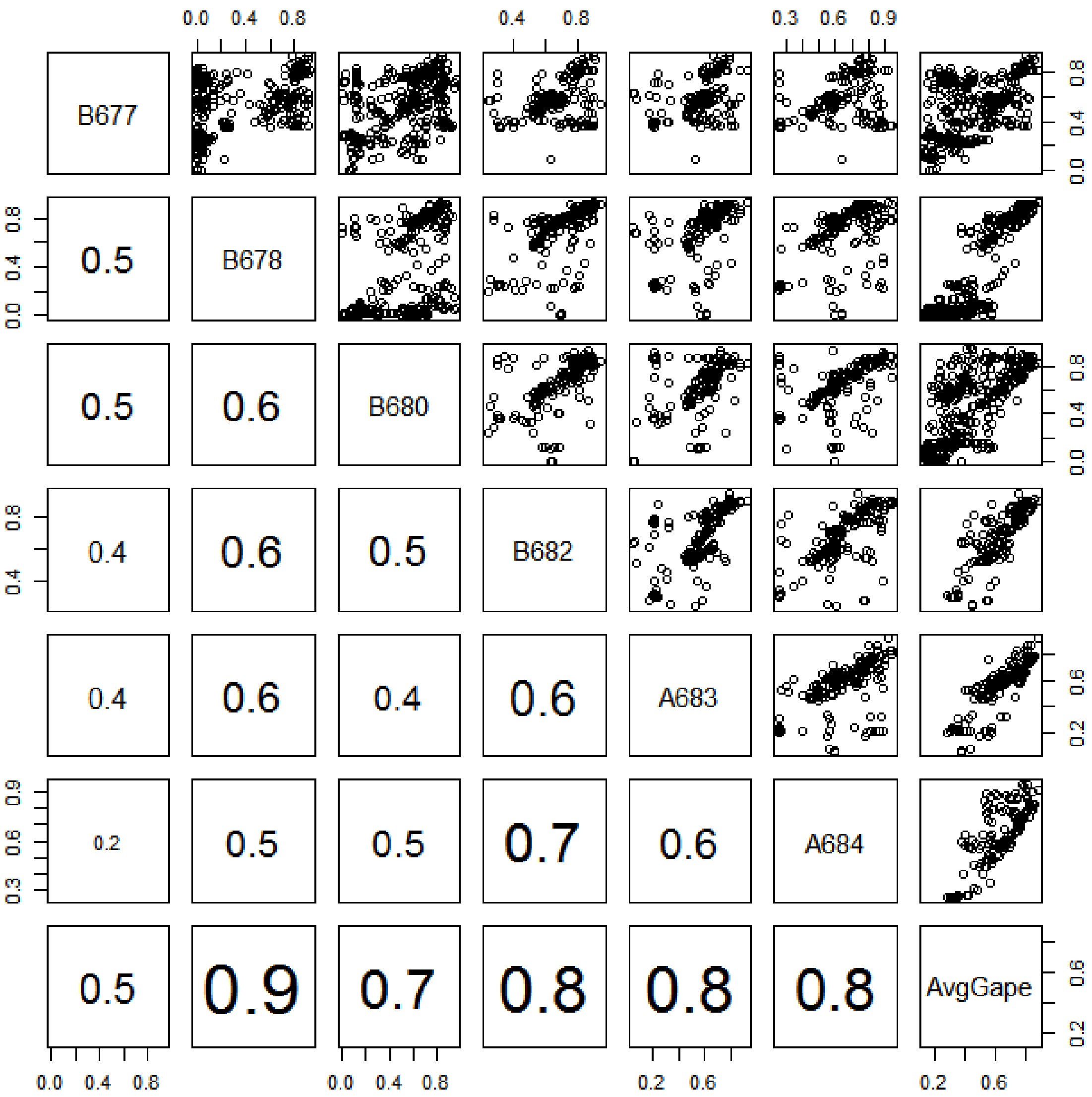

Fig. 6 Correlation plot between new specimens from recorder 2 and AvgGape. No data for B676 because the channel did not work

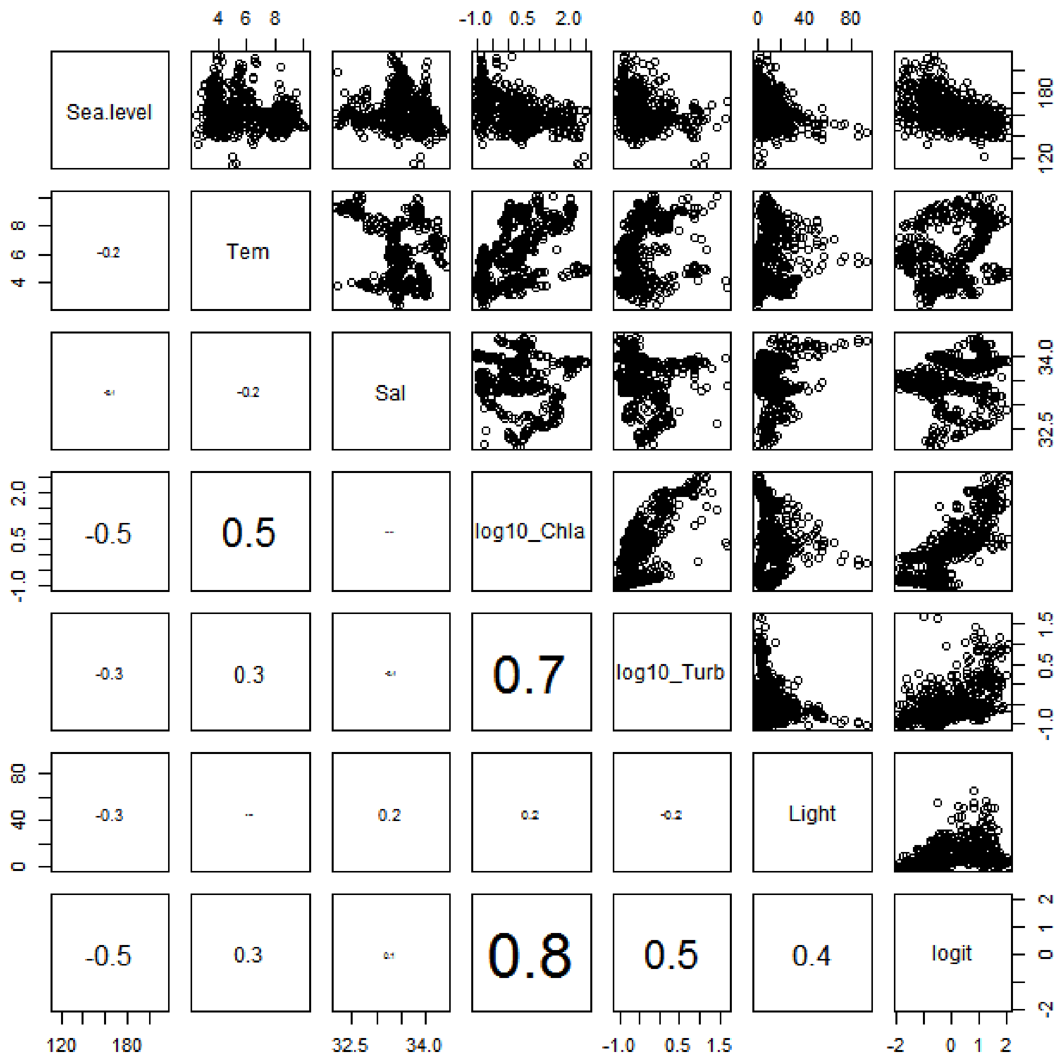

Fig. 7 Data exploration

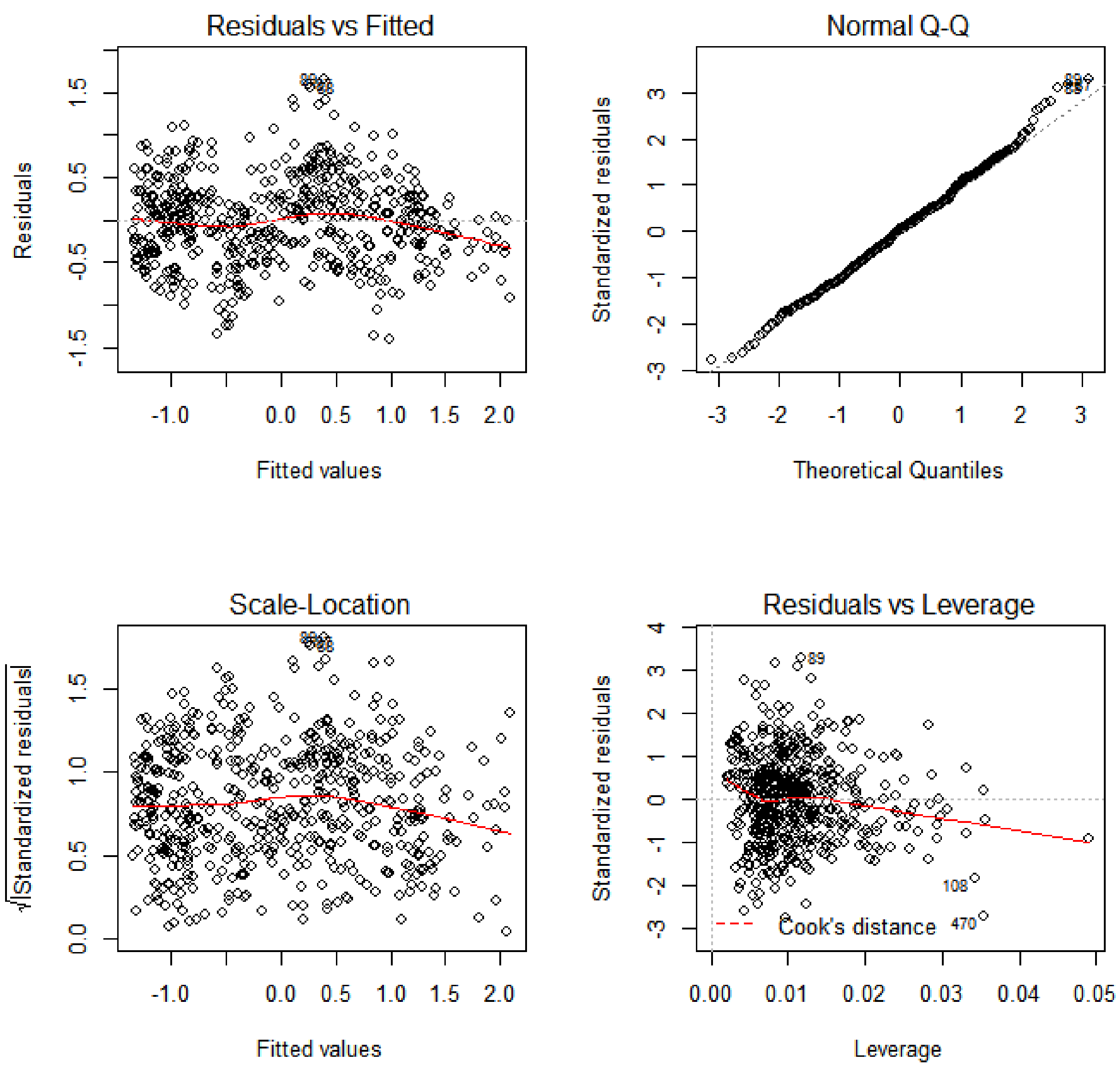

Fig. 8 Residuals plots for model M1

Table 1: Best regression model with one, two, three, four or five explanatory variables

Model	Variables	R^2-adjusted	F-statistic
**M1.1**	logit(AvgGape) ~log_Chla	0.6566	1047
**M1.2**	logit(AvgGape) ~log_Chla + Light	0.7162	691.1
**M1.3**	logit(AvgGape) ~log_Chla + Light + Tem	0.7435	529.6
**M1.4**	logit(AvgGape) ~log_Chla + Light + Tem+ Sal	0.7461	402.8
**M1**	logit(AvgGape) ~log_Chla + Light + Tem+ Sal+Sea level	**0.7497**	**328**

Table 2: Regression table for model M2

M2 logit(AvgGape) ~	Coeffcient	Std.Error	t-value	P-value
**(Intercept)**	-0.01497	0.02176	-0.688	0.4919
**PC1**	0.48232	0.01429	33.755	<2E-16
**PC2**	0.26014	0.01862	13.969	<2E-16
**PC3**	0.10709	0.02202	4.863	1.52E-16
**PC4**	0.06742	0.02758	2.445	0.0148
**PC5**	0.34796	0.02955	11.776	<2E-16
**PC6**	-0.62921	0.5412	-11.625	<2E-16
			**R^2-adjusted=**	**0.75**
